# Implementation of 26 Gy in five fractions over 1 week adjuvant radiotherapy for breast cancer: Prospective report of acute skin toxicity and consideration of resource implications^☆^

**DOI:** 10.1016/j.breast.2022.12.008

**Published:** 2022-12-16

**Authors:** K. Nugent, E. Quinlan, S. Cleary, H. O'Driscoll, C. Rohan, J. Trousdell, J. Williams, M. Dunne, O McArdle, F.K. Duane

**Affiliations:** a St. Luke's Radiation Oncology Network, St. Luke's Hospital, Dublin, Ireland; b Trinity St James's Cancer Institute, St. James's Hospital, Dublin, Ireland; c School of Medicine, Trinity College Dublin, Ireland

**Keywords:** Breast cancer, Adjuvant radiotherapy, Ultrafractionation, Acute toxicity, Resource implications

## Abstract

**Purpose:**

In March 2020, a 1-week adjuvant breast radiotherapy schedule, 26 Gy in 5 fractions, was adopted to reduce the risk of COVID19 for staff and patients. This study quantifies acute toxicity rates and the effect on linac capacity.

**Materials and methods:**

This is a report of consecutive patients receiving ultrafractionated breast radiotherapy ( ± sequential boost) Mar–Aug 2020. Virtual consultations assessed acute skin toxicity during treatment and weeks 1, 2, 3 and 4 post treatment using CTCAE V5 scoring criteria. The number of linac minutes saved was estimated accounting for boost and DIBH use.

**Results:**

In total, 128/135 (95%) patients, including 31/33 boost patients, completed at least 3/5 assessments. 0/128 (0%) reported moist desquamation not confined to skin folds or minor bleeding (Grade 3), 41/128 (32%) reported brisk erythema, moist desquamation confined to skin folds or breast swelling (Grade 2), 62/128 (48%) reported faint erythema or dry desquamation (Grade 1) as their worst skin toxicity, with the remaining 20% reporting no skin toxicity. The highest prevalence of grade 2 toxicity occurred week 1 following treatment (20%), reducing to 3% by week 4. There was no difference in toxicity between those who received a boost versus not (p = 1.00). Delivering this schedule to 135 patients over six months saved 21,300 linac minutes and 1485 hospital visits compared to a 3-week schedule.

**Conclusion:**

Rapidly implementing ultrahypofractionated breast radiotherapy is feasible and acute toxicity rates are acceptable even when followed by boost.

## Highlights

• It is feasible and safe to rapidly implement ultrahypofractionated breast RT.
• 26 Gy in 5 fractions ( ± sequential boost) leads to G0-2 acute skin toxicity.
• G3 acute skin toxicity is usually not observed with ultrahypofractionated breast RT.
• Ultrahypofractionation does not lead to excess toxicity when breast volume is large.
• Ultrahypofractionation saved 6300 LINAC minutes over 6 months (135 patients).

## 1 Introduction

The COVID 19 (SARS-CoV-2) health crisis led radiation oncologists to change clinical practice where possible in an effort to reduce patients’ exposure to the virus. Expeditious implementation of safe and effective hypofractioned schedules and standardised virtual consultations were adopted as a strategy to optimise radiotherapy linear accelerator capacity and reduce patient hospital visits. As adjuvant breast cancer radiotherapy can account for a significant proportion of fractions delivered in a radiation department shortening treatment schedules was likely to have a substantial positive impact on resources. In March–April 2020 national and international guidelines endorsed the use of a one week radiotherapy schedule, 26 Gy in 5 fractions, for patients with node-negative breast cancer [1,2]. Implementation of this 1-week schedule represented a landmark change in practice for radiation oncology in Ireland as the standard of care for whole breast radiotherapy was to deliver a moderately hypofractionated three-week schedule, 40 Gy in 15 fractions [3,4]. The phase III Fast-Forward clinical trial was subsequently published in May 2020 confirming equivalent breast cancer outcomes and normal tissue effects at 5 years [5].

Before the COVID-19 pandemic it was common clinical practice to deliver a hypofractionated boost following moderately hypofractionated whole breast radiotherapy. In 2019 the Royal College of Radiologists (RCR) UK suggested the use of hypofractionated boost regimens biologically equivalent to 16 Gy in 8 fractions such as 13.35 Gy in five fractions or 12 Gy in four fractions to the tumour bed following 40 Gy in 15 fractions to the whole breast [6] In 2018 ASTRO suggested the use of 10 Gy in four fractions, regardless of whole-breast fractionation schedule being used [7]. In the Fast-Forward trial, which investigated the effects of a 1-week schedule, hypofractionated boosts were not included in the protocol but 25% of patients underwent a sequential boost of 16 Gy in 8 fractions or 10 Gy in 5 fractions. Therefore, data are lacking on the effects of delivering a hypofractionated boost following a 1-week schedule. In Ireland, the continued use of a boost to the primary tumour bed during the COVID 19 pandemic was considered for patients aged <50 years or with a positive margin, and for patients aged 50–60 years with high grade disease, lymphovascular invasion or the presence of extensive intraductal component [2].

The benefit of hypofractionated schedules has been demonstrated in terms of improved cost-effectiveness [8] and patient convenience [9]. Models reviewing time, labour costs, and capital costs of moderately hypofractionated breast treatment schedules have shown a reduced financial cost and consumption of fewer resources associated with shorter treatment schedules [10,11]. Now real-world implications of using of a 1-week adjuvant schedule are of interest. The impact of implementing a 1-week schedule for a common disease such as breast cancer is likely to have major implications for future resource allocation and planning within radiotherapy departments as well as having a positive impact on infection control while the COVID-19 pandemic is ongoing.

This study aims to report the feasibility of implementing a 1-week adjuvant breast radiotherapy schedule rapidly across multiple centers and to confirm acceptable acute toxicity rates ( ± hypofractionated sequential tumour bed boost). It also quantifies the positive impact this landmark change in practice has had on linac capacity.

## 2 Methods

### 2.1 Implementation of 1-week dose fractionation schedule

Nine radiation oncologists from the St. Luke's Radiation Oncology Network in Dublin participating in breast cancer multidisciplinary team meetings at four university hospitals across Dublin city convened and agreed on the implementation of a 1-week schedule, 26 Gy in 5 fractions in March 2020. Eligibility criteria were established [2]. Consensus was achieved on a treatment planning protocol in conjunction with physicists and dosimetrists which included dose volume evaluation and mandatory radiation quality assurance objectives (Supplementary Fig. 1). The sequential boost dose fractionation schedule was at the discretion of the treating radiation oncologist (Table 1). A comprehensive patient follow-up schedule was offered to ensure safe practice. All consecutive patients receiving 26 Gy in 5 fractions adjuvant breast cancer radiotherapy ( ± boost) March–August 2020 were included. This work was not regarded as a clinical trial. The 1-week ultrahypofractionated schedule was discussed with and offered routinely to all patients meeting the eligibility criteria in the clinic. A prospective database was set up to record toxicities. The reporting of the results presented was approved by the Research Ethics Committee, St. Luke's Radiation Oncology Network. All patients provided written informed consent for ultrahypofractionated radiotherapy and follow up was offered to all patients but was optional.

**Table 1:** Patient, tumour and treatment variables among patients who received 26 Gy in 5 fractions adjuvant breast cancer radiotherapy March-August 2020.

Variable	No patients	(%)
**(a)**Patient		
**Age, years at diagnosis**		
<40	1	1
40–49	7	5
50–59	33	24
60–69	50	37
70–79	39	29
80+	5	4
**(b)**Tumour		
**Breast cancer laterality**		
Left	64	47
Right	71	53
**Histology Type**		
Ductal	105	78
Lobular	11	8
Mixed	8	6
Other	11	8
**Tumour stage**		
Tis	2	2
T1	91	67
T2	41	30
T3	0	0
T4	1	1
**Number of positive nodes**		
pN0	117	87
pN1 (mi)	12	9
pN1a	5	3
pN2	1	1
**Oestrogen receptor status**		
Positive	126	93
Negative	9	7
**Her 2 receptor status**		
Positive	12	9
Negative	123	91
**Tumour Grade**		
Grade 1	27	20
Grade 2	73	54
Grade 3	35	26
**Lymphovascular invasion**		
Absent	97	72
Present	38	18
**(c)**Treatment		
**Type of surgery**		
Mastectomy	5	4
Breast conserving	130	96
**Axillary Staging**		
Sentinel node biopsy	133	98
Axillary clearance	1	1
No axillary surgery	1	1
**Radiotherapy**		
Whole breast only	97	72
Whole breast + sequential boost		
10.68 Gy/4 f	25	19
12 Gy/4 f	1	1
13.35 Gy/5 f	2	2
16 Gy/8 f	5	3
Chest wall	5	3
**Deep Inspiration Breath Hold**		
Yes	21	16
No	114	84
**Endocrine Therapy**		
**Yes**	126	93
**No**	9	7
**Chemotherapy**		
Neo-adjuvant	11	8
Adjuvant	14	10
None	110	82
**All women**	**135**	**100**

#### 2.1.1 Radiotherapy treatment planning

Patients underwent a non-contrast CT-planning scan in the supine position. Deep inspiratory breath hold technique was considered for all left-sided breast patients <60 years or breaching heart or lung constraints to minimize exposure of the heart and lungs. A provisional tangential field pair was selected by the radiation oncologist. Fields were arranged to cover the target and minimize dose to the normal tissues by manipulating the gantry and collimator angles and using multileaf collimators for shaping the fields. The ipsilateral lung and heart were mandatory organ at risk contours. The radiation dosimetrist started by weighting the two main tangential fields as standard and converted the 95% isodose line cropped 5 mm from lungs and skin surface to a structure. This field-based volume, although not a true PTV, was termed Breast PTV for dose evaluation and breast volume measurement. Segment fields were then added and the volume of 105% was minimized as far as practicably achievable. The dose prescription was according to ICRU 50 [12]. For patients prescribed a tumour bed boost a photon boost was delivered to a PTV boost eval (PTV boost structure cropped 5 mm from lungs and skin surface to a structure) A 3D-conformal field arrangement was favored but mini-tangents were also used. Daily image guidance using kV imaging or electronic portal image device was undertaken.

#### 2.1.2 Acute toxicity virtual consultations

Standardised virtual consultations were undertaken during treatment and at weeks 1, 2, 3 and 4 post treatment during which acute skin toxicity was recorded using CTCAE v5 [13] scoring criteria for radiation dermatitis (Supplementary Table 1). For patients receiving a boost, post treatment assessment began one week after completion of the boost. A questionnaire was developed to determine toxicity grade (G0-4) over the phone during each consultation (Supplementary Fig. 2). Questions were standardised with all patients asked the same questions and the questioner completing a form recording toxicity during the call.

#### 2.1.3 Analyses acute toxicity

The primary endpoint was the proportion of patients with grade ⩾3 toxicity at any time. Secondary endpoints included the worst acute skin toxicity experienced by patients, and the prevalence of toxicity at each time point. Only patients who completed at least 3 out of 5 toxicity assessments were included in the analyses. Acute toxicity reported by patients who received a boost was compared with those who did not. Chi square tests were used to test for a significant association between toxicity and the receipt of a sequential boost. Logistic regression was performed to assess the association between the variables T-stage, chemotherapy received, and dosimetric parameters (Breast PTV V95%, Breast PTV Dmax, Breast PTV V105% in cc, Body PTV V105% in cc, Breast PTV Volume cc, Boost PTV Eval Volume cc, and Boost PTV Dmax) and grade 2 toxicity. Mann-Whitney U tests were used to test for significant differences in the distribution of these dosimetric parameters and grade 2 toxicity. All statistical tests were two-sided and assessed for significance at the 0.05 level. Statistical analysis was carried out using IBM SPSS Statistics 25 (Chicago, IL).

### 2.2 Analyses resource implications and patient hospital attendance

The average number of linac minutes required for treatment delivery was determined according to the treatment received (1-week schedule, no DIBH; 1-week schedule using DIBH; 1-week schedule + boost, no DIBH; 1-week schedule + boost, DIBH) and assigned to each of the 135 patients enrolled on the study. The average total number of linac minutes saved was then calculated by subtracting the number of minutes required to deliver the 1-week schedule from the number that would have been required should a moderately hypofractionated schedule have been used. The number of hospital visits avoided by the adoption of the 1-week schedule was also determined.

## 3 Results

### 3.1 Patient population

Between March 2020 and August 2020, a 1-week adjuvant breast radiotherapy schedule, 26 Gy in 5 fractions was delivered to 135 women (Table 1). All patients completed the radiotherapy course prescribed. Thirty-seven percent of patients were aged 60–69 years and 33% were >70 years. Only 6% were aged <50 years. 97% had T1-2 primary lesions, 74% were G1-2, 18% had LVI and 93% were oestrogen receptor positive. Five patients (4%) underwent a mastectomy. Deep inspiratory breath hold was used in 21 patients (16%). Thirty-three (25%) received a sequential photon boost. The dose fractionation schedules used were 10.68 Gy in 4 fractions (25/33 patients), 12 Gy in 4 fractions (1/33), 13.35 Gy in 5 fractions 2/33 patients and 16 Gy in 8 fractions (5/33 patients). Most patients received adjuvant endocrine therapy (93%) with 18% of patients receiving chemotherapy in either the neo-adjuvant or adjuvant setting.

### 3.2 Radiotherapy quality assurance

All radiotherapy plans met all of the pre-determined planning objectives (Supplementary Table 2).

#### 3.2.1 Acute toxicity

**Fig. 1:**
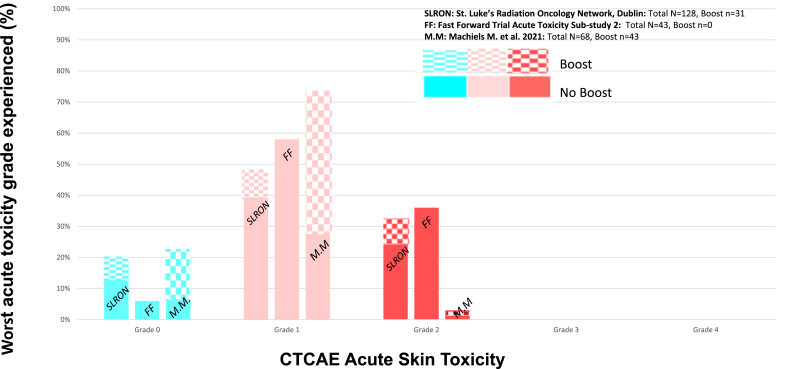
Worst acute skin toxicity reported following 26 Gy in 5 fractions to whole breast ( ± sequential boost) for 128 patients treated in the St. Luke's Radiation Oncology Network Dublin Mar–Aug 2020 and in previously published studies.

**Fig. 2:**
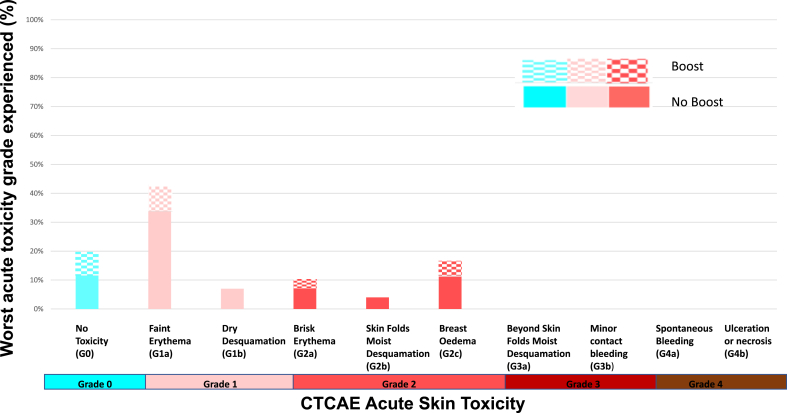
Worst acute skin toxicities reported following 26 Gy in 5 fractions to whole breast ( ± sequential boost) for 128 patients treated in the St. Luke's Radiation Oncology Network Dublin Mar–Aug 2020.

The worst acute skin toxicities reported were: G0: 25/128 (20%), G1: 62/128 (48%) G2: 41/128 (32%) and there were no G3 or G4 toxicities reported, consistent with previously published results (Fig. 1). There were no statistically significant differences in either G2 or G1-2 acute toxicity between the boost and no boost groups (p = 1.00 and p = 0.20 respectively). The worst acute skin toxicities reported for patients who underwent 26 Gy in 5 fractions versus 26 Gy in 5 fractions plus a boost respectively were: G0: 16/97 (17%) versus 9/31 (29%); G1: 51/97 (52%) versus 11/31 (35%); G2: 30/97 (30%) versus 11/31 (35%). Faint erythema (G1a) was the most common worst adverse effect experienced followed by breast oedema (G2c) regardless of the delivery of a boost (Fig. 2).

**Fig. 3:**
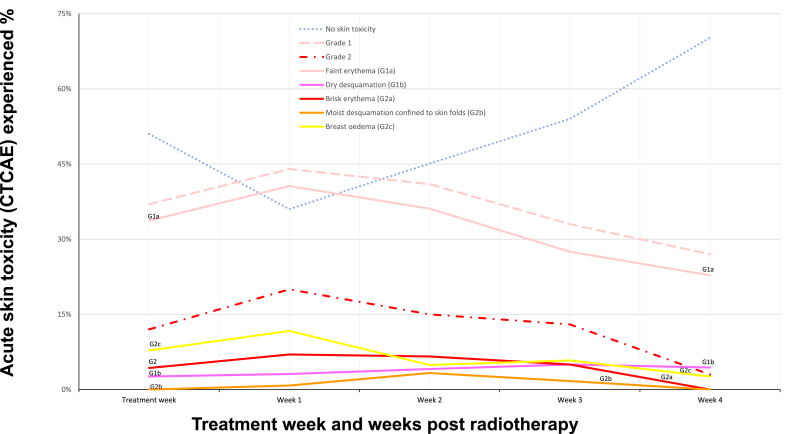
Acute skin toxicity reported following 26 Gy in 5 fractions to whole breast ( ± sequential boost) during the first week of treatment and weeks 1–4 following treatment for 128 patients treated in the St. Luke's Radiation Oncology Network Dublin Mar–Aug 2020. For patients receiving a boost, week 1 post treatment toxicity occurred one week after completion the boost.

The highest prevalence of both Grade 1 and 2 toxicities occurred at week 1 following treatment (Fig. 3). Faint erythema was the most common acute toxicity observed reported for 44% of patients week 1 after treatment and persisted at week 4 for 27% of patients. Grade 2 toxicity peaked at 20% week 1 but reduced to 3% by week 4.

In the logistic regression analysis, none of the independent variables were found to predict grade 2 toxicity when assessed individually (Table 2). Mann-Whitney U tests revealed no statistically significant difference between the distribution of individual dosimetric parameters (Breast PTV V95%, Breast PTV Dmax, Breast PTV V105% in cc, Body PTV V105% in cc, Breast PTV Volume cc, Boost PTV Eval Volume cc, and Boost PTV Dmax) and grade 2 toxicity.

**Table 2:** Logistical regression analysis on the association of T stage, chemotherapy received and dosimetric parameters with grade 2 toxicity.

Variable	p	Odds Ratio	95% Confidence Interval for Odds Ratio	
			Lower	Upper
T-stage	.455	.73	.32	1.66
Chemotherapy received	.122	.40	.13	1.27
Breast PTV V95%	.515	1.06	.89	1.25
Breast PTV DMAX	.169	1.32	.89	1.97
Breast PTV V105 in cc	.081	1.03	1.00	1.05
Body PTV V105 in cc	.079	1.02	1.00	1.05
Breast PTV Volume cc	.464	1.00	1.00	1.00
Boost PTV Eval Volume cc	.119	.96	.92	1.01
Boost PTV DMAX	.993	.00	.00	

### 3.3 Resource implications and patient hospital attendance

**Fig. 4:**
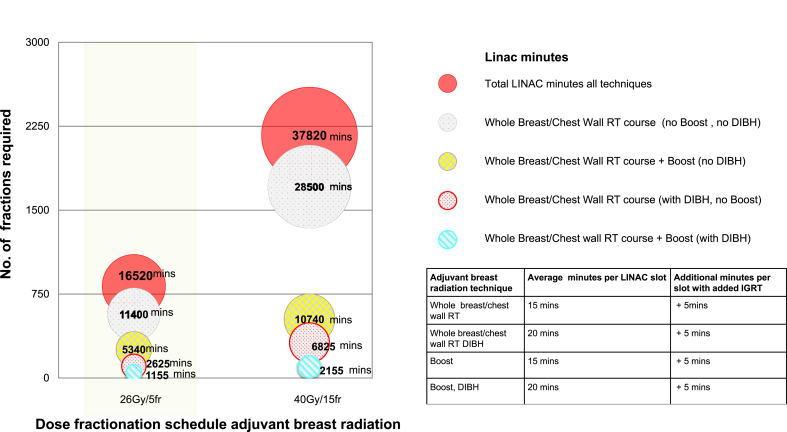
LINAC minutes saved using 26 Gy in 5 fractions for 135 patients treated in the St. Luke's Radiation Oncology Network, Dublin Mar–Aug 2020.

Delivering a 1-week schedule to 135 patients over a six-month period led to a saving of 21,300 linac minutes and 1485 hospital visits compared to delivering a moderately hypofractionated regimen of 3 weeks duration (Fig. 4). For those patients >70 years of age, who at that time, were advised to cocoon at home under national COVID pandemic guidelines, 6300 linac minutes were saved and 462 hospital visits avoided.

## 4 Discussion

This report outlines the feasibility of rapidly implementing a 1-week adjuvant breast radiotherapy schedule and an adjuvant breast radiotherapy virtual clinic across multiple sites. The results confirm acceptable acute toxicity using 26 Gy in 5 fractions over 1 week, with or without the addition of a sequential hypofractionated boost. 68% of patients experienced no or at most grade 1 acute toxicity. No grade 3/4 toxicity occurred during the follow up period. Faint erythema was the most common worst toxicity experienced by patients followed by breast oedema. Grade 2 toxicity peaked at 20% one week after radiotherapy and tapered to 3% by the fourth week following treatment completion. Tumour-related, treatment-related and dosimetry-related variables were not associated with increased risk of grade 2 toxicity. During the peak of the COVID 19 pandemic when reduced risk of exposure to the virus was considered of paramount importance especially in older patients, ultrahypofractionated radiotherapy regimens were widely adopted. Our findings regarding acute toxicity rates in this setting are reassuring.

There was a significant reduction in linac time utilisation and patient hospital visits during the period of implementation with an overall saving of 21,300 linac minutes and 1485 hospital visits for 135 patients treated over a 6-month period. In the initial phase of the COVID 19 pandemic, we had significant concerns regarding potential exposure of our patients to the virus in the healthcare environment. In addition, we were concerned that staff sick leave would result in an inability to maintain routine treatment workloads. Linac minutes and total hospital visits were metrics captured to give some data regarding the reduced risk to patients by reduced attendance and the mitigation of potential staffing issues by reducing treatment time requirements on linacs. A wider analysis of the economic impact of introducing ultrahypofractionated radiotherapy for the treatment of breast cancer is outside the scope of this paper, but is an important question given the large proportion of radiotherapy workload represented by adjuvant breast cancer radiotherapy treatments.

Our study has several strengths. Data were recorded prospectively with a high compliance rate for follow up with 95% of patients completing at least three standardised follow ups. A weekly follow-up schedule allowed a reporting of the timing of the worst radiation reactions. A potential weakness was the use of phone follow up assessments in lieu of face to face physician assessments. However, recent studies suggest patients’ experience and perception of telemedicine clinics during the COVID19 pandemic is as good as their traditional face-to-face experience [14] and there is longstanding evidence regarding the validity of patient reported outcomes in the oncology literature [15]. Virtual consultations were standardised with all patients asked the same questions and the questioner completing a form recording toxicity during the call. Video consultations were not available to us in the initial phase of the pandemic. Interestingly, we have since found in a further study of patient reported experience measures after breast radiotherapy that just over half the women asked reported that they would not be comfortable with a physician carrying out a skin/breast review by video call [16].

Our acute toxicity results are similar to the FAST-Forward acute toxicity sub study 2 (FF sub-study 2) [17] which also used CTCAE scoring criteria [13], demonstrating that grade 1 toxicity was the most commonly reported toxicity in 43 patients treated with 26Gy in 5 fractions and no boost. Our results are also comparable to those of a prospective observational study by Machiels et al. [18], including 68 patients treated with the one-week regimen, 43 of whom also received a boost of 6Gy in a single fraction. 74% of patients reported grade 1 dermatitis as their worst acute toxicity, 3% experienced grade 2 toxicity, none experienced grade 3 toxicity. As in our study, there was no significant toxicity difference between patients who had received the boost. In our study, a specific sequential boost dose fractionation schedule was not mandated; 10.68Gy in 4 fractions was the most commonly used regimen, given to 25 of 33 women who received a boost. Currently, there is no high-level evidence supporting one schedule over another but even before the COVID-19 pandemic it was common clinical practice to deliver a hypofractionated boost following moderately hypofractionated whole breast radiotherapy. The low grades of acute skin toxicity reported in these studies are not unexpected. The weak dependence of erythema and desquamation on fraction size is known and lower acute toxicity is expected with a lower total biological equivalent dose of radiation [19,20].

The timing of worst acute toxicity is likely different for ultrahypofractionated versus moderately hypofractionated radiotherapy. Our data and previously published studies suggest the highest prevalence of toxicity after 26Gy in 5 fractions in one week occurs in the first two weeks after treatment [17,18]. We found 70% of women had no toxicity by week 4 of follow up. Prospective studies of toxicity after moderately hypofractionated radiotherapy (40Gy in 15 fractions) demonstrate peak incidence of grade 2 skin reactions at week 5 and week 4 respectively [17,21]. These findings provide clinicians with more accurate information on which to base advice regarding follow up care after differing radiation treatment schedules.

Breast volume, boost volume, chemotherapy treatment and dosimetry metrics were not statistically significant predictors of increased toxicity. Concerns regarding increased toxicity associated with increased breast size were not supported by our findings suggesting that large breast volumes can be safely treated once planning objectives are within constraints.

Our study demonstrates real world implications for linac capacity of implementing the 1-week schedule. There was a significant reduction in the need for patient hospital attendance and reduced risk of COVID19 exposure for staff and patients during the peak of the pandemic. This strategy was particularly beneficial to patients over the age of 70 who were cocooning in accordance with national guidelines at the time. The scale of reduction of linac minutes was large.Delivering this schedule to 135 patients over six months saved 21,300 linac minutes and 1485 hospital visits compared to a 3-week schedule. This represented a significant reduction in workload in a department facing staffing challenges during the pandemic. Beyond mitigating the risk of COVID 19, the adoption of a shorter treatment schedule for adjuvant breast cancer patients has major resource implications in terms of reduced financial costs and consumption of labour [10]. Its continued adoption and implementation will positively impact future radiation capacity planning, resource allocation and financial costs.

While data regarding the acute toxicity of ultrahypofractionation is reassuring, fraction size can heavily influence late tissue effects. Both the long-term follow-up from the moderately hypofractionated trials [4] and the recent FAST-Forward trial have not shown increased rates of skin telangiectasia, breast fibrosis or reported adverse cosmetic outcomes, and have demonstrated non inferior local recurrence rates [5]. This suggests that breast cancer and late effects in breast tissue have similar responses to large doses per fraction radiation. Confirmatory studies reviewing the late breast tissue effects should be carried out to ensure no unacceptable increase in rates of late skin toxicity or deteriorations in patient reported cosmetic outcomes. Future late toxicity studies including the addition of a sequential hypofractionated boost or synchronous integrated boost will be of interest.

In conclusion, this study demonstrates the feasibility of rapidly implementing a 1 week ultrahypofractionated adjuvant breast radiotherapy schedule in clinical practice and how this landmark change has a considerable impact on linac capacity. This change in practice has ensured ongoing access to treatment for patients during the COVID-19 pandemic and greatly reduced the risks of infection for patients and staff. It further confirms acceptable acute skin toxicity including when followed by boost.

## Declaration of competing interest

None.

## References

[1] Coles C.E., Aristei C., Bliss J., Boersma L., Brunt A.M., Chatterjee S. (2020). International guidelines on radiation therapy for breast cancer during the COVID-19 pandemic. Clin Oncol.

[2] Library H.S.E. HSE library guides: covid-19 HSE clinical guidance and evidence: radiation oncology - NCCP advice for medical professionals on the management of patients undergoing breast cancer radiotherapy in response to COVID-19 n.d. https://hse.drsteevenslibrary.ie/c.php?g=679077&p=4865893.

[3] Haviland J.S., Owen J.R., Dewar J.A., Agrawal R.K., Barrett J., Barrett-Lee P.J. (2013). The UK Standardisation of Breast Radiotherapy (START) trials of radiotherapy hypofractionation for treatment of early breast cancer: 10-year follow-up results of two randomised controlled trials. Lancet Oncol.

[4] Whelan T.J., Pignol J.-P., Levine M.N., Julian J.A., MacKenzie R., Parpia S. (2010). Long-term results of hypofractionated radiation therapy for breast cancer. N Engl J Med.

[5] Murray Brunt A., Haviland J.S., Wheatley D.A., Sydenham M.A., Alhasso A., Bloomfield D.J. (2020). Hypofractionated breast radiotherapy for 1 week versus 3 weeks (FAST-Forward): 5-year efficacy and late normal tissue effects results from a multicentre, non-inferiority, randomised, phase 3 trial. Lancet.

[6] Radiotherapy dose fractionation, third ed. - breast cancer n.d.:8.

[7] Smith B.D., Bellon J.R., Blitzblau R., Freedman G., Haffty B., Hahn C. (2018). Radiation therapy for the whole breast: executive summary of an American Society for Radiation Oncology (ASTRO) evidence-based guideline. Pract Radiat Oncol.

[8] Bekelman J.E., Sylwestrzak G., Barron J., Liu J., Epstein A.J., Freedman G. (2014). Uptake and costs of hypofractionated vs conventional whole breast irradiation after breast conserving surgery in the United States, 2008–2013. JAMA.

[9] Shaitelman S.F., Schlembach P.J., Arzu I., Ballo M., Bloom E.S., Buchholz D. (2015). Acute and short-term toxic effects of conventionally fractionated vs hypofractionated whole-breast irradiation: a randomized clinical trial. JAMA Oncol.

[10] Dziemianowicz M., Burmeister J., Dominello M. (2021). Examining the financial impact of altered fractionation in breast cancer: an analysis using time-driven activity-based costing. Pract Radiat Oncol.

[11] Yaremko H.L., Locke G.E., Chow R., Lock M., Dinniwell R., Yaremko B.P. (2021). Cost minimization analysis of hypofractionated radiotherapy. Curr Oncol Tor Ont.

[12] Landberg T., Chavaudra J., Dobbs J., Hanks G., Johansson K.-A., Möller T. (1993). Report 50. J Int Comm Radiat Units Meas.

[13] (2017). Common terminology criteria for adverse events.

[14] Isautier J.M., Copp T., Ayre J., Cvejic E., Meyerowitz-Katz G., Batcup C. (2020). People's experiences and satisfaction with telehealth during the COVID-19 pandemic in Australia: cross-sectional survey study. J Med Internet Res.

[15] Bhattacharya I.S., Haviland J.S., Hopwood P., Coles C.E., Yarnold J.R., Bliss J.M. (2019). Can patient-reported outcomes be used instead of clinician-reported outcomes and photographs as primary endpoints of late normal tissue effects in breast radiotherapy trials? Results from the IMPORT LOW trial. Radiother Oncol.

[16] Nicholson J., Nugent K., Farmer G., Monaghan O., O'Driscoll H., Cleary S. (2022). One-Year toxicity of ultrahypofractionated breast radiotherapy (+/- sequential boost) and a survey of patient experience. Int J Radiat Oncol Biol Phys.

[17] Brunt A.M., Wheatley D., Yarnold J., Somaiah N., Kelly S., Harnett A. (2016). Acute skin toxicity associated with a 1-week schedule of whole breast radiotherapy compared with a standard 3-week regimen delivered in the UK FAST-Forward Trial. Radiother Oncol J Eur Soc Ther Radiol Oncol.

[18] Machiels M., Weytjens R., Bauwens W., Vingerhoed W., Billiet C., Huget P. (2020). Accelerated adaptation of ultrahypofractionated radiation therapy for breast cancer at the time of the COVID-19 pandemic. Clin Oncol R Coll Radiol G B.

[19] Turesson I., Thames H.D. (1989). Repair capacity and kinetics of human skin during fractionated radiotherapy: erythema, desquamation, and telangiectasia after 3 and 5 year's follow-up. Radiother Oncol J Eur Soc Ther Radiol Oncol.

[20] Yarnold J., Bentzen S.M., Coles C., Haviland J. (2011). Hypofractionated whole-breast radiotherapy for women with early breast cancer: myths and realities. Int J Radiat Oncol Biol Phys.

[21] Arsenault J., Parpia S., Goldberg M., Rakovitch E., Reiter H., Doherty M. (2020). Acute toxicity and quality of life of hypofractionated radiation therapy for breast cancer. Int J Radiat Oncol.

