# Supplementary material for: Implementation of 26 Gy in five fractions over 1 week adjuvant radiotherapy for breast cancer: Prospective report of acute skin toxicity and consideration of resource implications
Source: Breast. 2022 Dec 16;67:55–61. doi: 10.1016/j.breast.2022.12.008 (PMC9756602; doi:10.1016/j.breast.2022.12.008)
Supplement: Multimedia component 1 [file mmc1.pdf]

Supplementary Fig. 1. Dose Volume Evaluation 26 Gy in 5 fractions Whole Breast[1,2]

Dose Volume Evaluation 26 Gy in 5 fractions Whole Breast

Dose	95%	107%
26	24.7	27..82

Dose PTV	Fractions
26Gy	5

EVALUATION OF TARGET- Whole breast				
ENSURE TRUE TARGET COVERAGE IS ADEQUATE BY VISUALLY ASSESSING EACH AXIAL CT SLICE AS STANDARD				
**Volume**	**Parameter**	**Optimal**	**Mandatory**	**Result**
**Breast PTV***	$V_{95\%}$<br>$V_{105\%}$<br>$V_{107\%}$<br>D_{max}	≥ 95%<br>≤ 5%	≥ 90%<br>≤ 7%<br>≤ 2%<br>≤ 110%	%<br>%<br>%<br>%

*A field-based Breast PTV is generated by radiation therapists before any field segments are added. The Breast PTV is not a true PTV but a structure encompassing the 95% isodose line, which is cropped 0.5cm from lungs and skin surface. This is to be used for DVH evaluation.

EVALUATION OF OARs(13)				
**OAR**	**Parameter**	**Optimal Constraint**	**Mandatory**	**Result**
**Ipsilateral Lung**	$V_{30\%}$	≤ 15%	≤ 17%	%
**Heart**	$V_{5\%}$<br>$V_{25\%}$		≤ 25%<br>≤ 5%	%<br>%
**If Electronic device present**			D max	Gy

1. FAST-Forward Protocol Version 3.0: 08/07/2015

2. Planning Pack for the FAST-Forward Trial. Version 3

Accessed March 2020

Available at:https://www.icr.ac.uk/our-research/centres-and-collaborations/centres-at-the-icr/clinical-trials-and-statistics-unit/clinical-trials/fast_forward_page

Supplementary Table 1: Radiation Dermatitis Scoring Criteria for Adverse Event (CTCAE) v.5

	None	1	2	3	4
Radiation Dermatitis		1a: Faint Erythema<br><br>1b: Dry desquamation	2a:Moderate to brisk erythema, mostly in skin folds<br><br>2b: Moderate oedema	3a Confluent moist desquamation, other than skin folds<br><br>3b Bleeding induced by minor trauma	4a Skin necrosis or ulceration of full thickness dermis;<br>4b Spontaneous bleeding from involved site;<br>4c Skin graft indicated

Supplementary Figure 2: Virtual Consultation

ACUTE TOXICITY ASSESSMENT							
Date Last Treatment :							
Patient’s Assessment: Questions are in relation to how their skin has been in the last 7 days							
Weeks post treatment:	**Base line**	**1**	**2**	**3**	**4**		
Today’s Date:							
Has your skin changed colour?						No	Grade 0
						Yes	Grade 1a = Faint red / pink<br>Grade 2a = Bright red
Is your skin dry, flaky, or scaly?						Yes	Grade 1b
Are there moist or wet areas of skin breakdown?						Yes	Grade 2b = confined to skin folds<br>Grade 3a = other areas
Does your skin bleed?						Yes	Grade 3b = minor trauma<br>Grade 4a = spontaneously
Is your breast swollen						Yes	At least grade 2c
Are there areas of deep aggressive ulceration?<br>Are there areas of muscle on show?						Yes	Grade 4b

Supplementary Table 2 Radiation Quality Assurance- Analysis of radiation planning objectives for the 135 breast patients who underwent ultrafractionation at our Radiation Network (March – August 2020)

	Breast PTV V95%	Breast PTV DMAX	Breast PTV V105%	Body V105%	Body V105% (cc)	Heart V5%	Heart V25%	i/l lung V30%
Median	96.3	105.5	0.1	0	0.2	2.1	0	11.5
Min	90.1	104	0	0	0	0	0	2.4
Max	99.8	109.2	4.8	0.2	109.2	24.8	5	17
